# Trends in the use of cigarettes, cigars, and marijuana among students with and without asthma, 2003–2017

**DOI:** 10.1080/02770903.2019.1573254

**Published:** 2019-02-07

**Authors:** Sherry Everett Jones, Brian A. King, Zanie Leroy

**Affiliations:** a Division of Adolescent and School Health, National Center for HIV/AIDS, Viral Hepatitis, STD, and TB Prevention, Centers for Disease Control and Prevention, Atlanta, GA, USA;; b Office on Smoking and Health, National Center for Chronic Disease Prevention and Health Promotion, Centers for Disease Control and Prevention, Atlanta, GA, USA;; c Division of Population Health, National Center for Chronic Disease Prevention and Health Promotion, Centers for Disease Control and Prevention, Atlanta, GA, USA

**Keywords:** Tobacco use, cigarette smoking, cigar smoking, marijuana use, school health services, high school students

## Abstract

**Aim:**

Asthma has symptoms that can be exacerbated by using combustible products such as tobacco and marijuana. This study assessed the prevalence and trends in current use of cigarettes, cigars, and marijuana among U.S. high school students with and without asthma.

**Methods:**

The national Youth Risk Behavior Survey (YRBS) is a biennial, school-based, nationally representative survey of U.S. students in grades 9–12. Trends during 2003–2017 in current (past 30-day) use of cigarettes, cigars, and marijuana among students with and without asthma were examined using logistic regression. Asthma was defined as reporting that a doctor or nurse ever told the student they had asthma. T-tests were used to compare cigarette, cigar, and marijuana use by asthma status and year, frequency of use, and student characteristics.

**Results:**

During 2003–2017, both current cigarette and cigar smoking decreased (p < 0.05) with rates that were similar among students with and without asthma. There was no significant linear change in current marijuana use, irrespective of asthma status. During most years, cigarette, cigar, and marijuana use were more common among students with asthma than without. In 2017, the most frequent use of cigarettes, cigars, and marijuana was among those with asthma than without. Differences in cigarette, cigar, and marijuana use by asthma status were apparent by demographic subgroups.

**Conclusions:**

Given the adverse respiratory effects of tobacco and marijuana smoking, efforts to educate all youth about the dangers of using these substances is critical, particularly among those with asthma.

## Introduction

Asthma, a chronic lung disease, affects an estimated 25 million people in the US, of which an estimated 7 million are children [1]. The U.S. Surgeon General has concluded that there is sufficient evidence to infer a causal relationship between active cigarette smoking and asthma exacerbation among adults [2]. Given that tobacco smoking is typically first initiated during adolescence [3], and asthma is usually first observed during youth and young adulthood [1], active cigarette smoking during this period could adversely influence the clinical course of asthma shortly after its onset [2]. To date, there is suggestive evidence that active cigarette smoking is associated with incident asthma among adolescents, as well as the exacerbation of asthma among both children and adolescents [2]. Additionally, exposure to secondhand smoke causes more frequent and severe asthma among children [4]. It is hypothesized that tobacco smoking can result in chronic inflammation of the airways [2], increased mucus production [5] and a reduced ability to clear mucus [2] and other irritants found in tobacco smoke as a result of damage to the lung’s cilia [5], lungs that are more sensitive to irritants [2], and diminished growth of a young person’s developing lungs [2]. These factors can contribute to worsened asthma symptoms.

The effects of smoking marijuana on the symptoms and severity of asthma are less clear [6–8]. Smoking marijuana is the most common mode of use among youth [9,10] and is smoked in many forms, including hand-rolled cigarettes (joints), in pipes or water pipes (bongs), in bowls, or in blunts (emptied cigars that have been partly or completely refilled with marijuana) [11]. Researchers do not yet know the full extent of the consequences of marijuana use on the body and brain [8]. Marijuana smoke may be a bronchodilator for some people with asthma [6]; however, it has not been scientifically shown to have a therapeutic role for patients because of modest and inconsistent experiences among patients, as well as because marijuana use may have a wide range of adverse health effects, including respiratory effects [6,8,11]. Smoked marijuana, in any form, can harm lung tissues and cause scarring and damage to small blood vessels [11]. Smoking marijuana can also lead to a greater risk of bronchitis, cough, and phlegm production [6,7,11,12]. Furthermore, smoke from marijuana contains many of the same toxins, irritants, and carcinogens as tobacco smoke [11].

Cigarette and cigar smoking have significantly decreased during the past two decades among high school students nationwide [13]. The prevalence of past 30-day cigarette smoking significantly decreased from 28% to 9% during 1991–2017. Data on past 30-day cigar smoking was first available in 1997, and its prevalence significantly decreased from 22% to 8% during 1997–2017. In contrast, no significant decrease or increase in the prevalence of past 30-day marijuana use occurred among high school students during 1991 (14.7%) to 2017 (19.8%) [13]. Prior research has shown that young people with asthma are as likely or more likely to use various tobacco products than students without asthma, including cigarettes [14–17], cigars [14–16], pipes [15], electronic cigarettes [16], and hookah [16]. Additionally, young people with asthma have also been shown to be more likely to use marijuana [14] and inhalants [14] than those without asthma.

To date, no study has assessed trends in the use, or frequency in the use, of tobacco and marijuana among youth with and without asthma. To address this existing gap in the scientific literature, this study used nationally representative data to examine trends in current use of cigarettes, cigars, and marijuana during 2003–2017 among high school students with and without asthma. In addition, this study compared the prevalence and frequency of current use of cigarettes, cigars, and marijuana among students with and without asthma, and the prevalence of current use of cigarettes, cigars, and marijuana by asthma status among different sex, racial/ethnic, and school grade subgroups.

## Methods

### Data source

This study examined data from the national Youth Risk Behavior Survey (YRBS), a cross-sectional, school-based, pencil and paper survey that has been conducted by the Centers for Disease Control and Prevention (CDC) biennially since 1991 [13,18,19]. Each survey year, an independent three-stage, cluster sample design is used to obtain a nationally representative sample of public and private school students in grades 9–12 in the 50 U.S. states and District of Columbia. We analyzed data from the national 2003–2017 YRBS because asthma status was first included in the YRBS questionnaire in 2003. During 2003–2017, sample sizes ranged from 10 904 to 16 410. School response rates ranged from 75% to 81%, student response rates ranged from 81% to 88%, and overall response rates (the product of the school and student response rates for each year) ranged from 60% to 71%.

Student participation in the YRBS is anonymous and voluntary, and local parental permission procedures are used. Survey participants complete a self-administered questionnaire during a regular class period and record their responses on a computer-scannable answer sheet. The protocol for the national YRBS was approved by the Institutional Review Board at the CDC.

### Measures

#### Asthma status

Asthma status was assessed using the question: “Has a doctor or nurse ever told you that you have asthma?” Response options were “yes,” “no,” and “not sure.” Respondents who indicated “yes” were considered to have asthma, otherwise, respondents were coded as not having asthma (unweighted numbers of the “not sure” response comprised less than 5% of respondents).

#### Cigarette and cigar smoking

Current cigarette and cigar smoking was assessed using two questions: “During the past 30 days, on how many days did you smoke cigarettes?” and “During the past 30 days, on how many days did you smoke cigars, cigarillos, or little cigars?” Response options for both questions were as follows: “0 days,” “1 to 2 days,” “3 to 5 days,” “6 to 9 days,” “10 to 19 days,” “20 to 29 days,” and “all 30 days.” Respondents who indicated that they smoked cigarettes or cigars on one or more days during the 30 days before the survey were considered current cigarette and cigar smokers, respectively.

#### Marijuana use

Current marijuana use was assessed using the question, “During the past 30 days, how many times did you use marijuana?” Response options were “0 times,” “1 to 2 times,” “3 to 9 times,” “10 to 19 times,” “20 to 39 times,” and “40 or more times.” Respondents who indicated that they used marijuana one or more times during the 30 days before the survey were considered current marijuana users.

### Analysis

To account for the complex sample design of the survey, all analyses were conducted using SUDAAN, version 11.0.1. A weight based on student sex, race/ethnicity, and grade was applied to each record to adjust for school and student nonresponse and over-sampling of black and Hispanic students. Missing data were not imputed.

For each survey year, t-tests were used to determine differences in the prevalence and frequency of current use of cigarettes, cigars, and marijuana by asthma status, and for 2017, for each sex, racial/ethnic, and grade subgroup. To assess linear time effects in asthma status and in the current use of cigarettes, cigars, and marijuana during 2003–2017 among students with asthma and without asthma, we used logistic regression models that controlled for sex, race/ethnicity and grade. Linear time variables were treated as continuous and were coded using orthogonal coefficients. To examine whether linear trends in current use of cigarettes, cigars, and marijuana differed by asthma status, an interaction term (time by asthma status) was included in the logistic regression model. Multiple product use and combinations of cigarettes, cigars, and marijuana use were not assessed due to limited sample size. For all analyses, *p* < 0.05 was considered statistically significant.

## Results

### Demographic characteristics of use

In 2017, students with asthma had a significantly higher prevalence of current cigarette smoking (10.7% vs. 8.2%; *p* < 0.05), current cigar smoking (9.7% vs. 7.3%; *p* < 0.05), and current marijuana use (23.4% vs. 18.5%; *p* < 0.05) than those without asthma (Table 1). The prevalence of current cigarette smoking was significantly higher among students with asthma than among those without asthma among female students (10.1% vs. 7.1%; *p* < 0.05), white students (13.7% v. 10.3%; *p* < 0.05), and 11^th^ grade students (12.8% vs. 8.3%; *p* < 0.05). The prevalence of current cigar smoking was significantly higher among students with asthma than among those without asthma among female students (7.4% vs. 4.7%; *p* < 0.05) and 11^th^ grade students (11.7% vs. 8.2%; *p* < 0.05). The prevalence of current marijuana use was significantly higher among students with asthma than among those without asthma among female students (24.7% vs. 18.0%; *p* < 0.05), white (21.2% vs 16.6%; *p* < 0.05) and Hispanic (29.3% vs. 21.7%; *p* < 0.05) students, and students in all grade subgroups.

### Frequency of use

In 2017, there was some variation by asthma status in the number of days students smoked cigarettes and cigars and the number of times students used marijuana. The percentage of students who smoked cigarettes and smoked cigars on 0 of the 30 days before the survey, and the percentage who used marijuana 0 times during the 30 days before the survey, was significantly higher among students without asthma than among students with asthma: 91.8% vs. 89.3% for cigarettes, 92.7% vs. 90.3% for cigars, and 81.5% vs. 76.6% for marijuana (all *p* < 0.05) (Table 2). In contrast, the percentage of students who smoked cigarettes on 3 to 5 days (1.7% vs. 1.2%; *p* < 0.05) and all 30 days (3.2% vs. 1.7%; *p* < 0.05), and the percentage of students who smoked cigars on 6 to 9 days (1.6% vs. 0.9%) and all 30 days (1.7% vs. 0.7%; *p* < 0.05) was significantly higher among students with asthma than among students without asthma. Similarly, the percentage of students who used marijuana 20–39 times (2.7% vs. 1.5%; *p* < 0.05) and 40 or more times (5.2% vs. 3.5%; *p* < 0.05) was significantly higher among students with asthma than among students without asthma.

### Trends in use

The prevalence of current cigarette smoking was significantly higher among students with asthma than without asthma for all years (all *p* < 0.05) except 2005 and 2007, when there were no significant differences. The prevalence of current cigar smoking was significantly higher among students with asthma than without asthma for all years (all *p* < 0.05) except 2007, when there was not a significant difference. The prevalence of current marijuana use was significantly higher among students with asthma than without asthma for all years (all *p* < 0.05) except 2005, when there was not a significant difference.

Logistic regression models indicate significant linear declines in current cigarette smoking and current cigar smoking among students with and without asthma during 2003–2017 (both *p* < 0.05) (Table 3). Current cigarette smoking significantly decreased from 25.2% to 10.7% among students with asthma, and from 22.1% to 8.2% among students without asthma. Current cigar smoking significantly decreased from 15.8% to 9.7% among students with asthma, and from 13.8% to 7.3% among students without asthma. No significant interaction was observed by time and asthma status. No significant linear change in current marijuana use was observed during 2003–2017 for either students with asthma or those without asthma.

## Discussion

The findings from this study reveal that although the prevalence of cigarette and cigar smoking declined among students with asthma during 2003–2017, the rate of decline was not significantly different among students with asthma compared to students without asthma. The prevalence of marijuana use remained unchanged during 2003–2017 among students with and without asthma. Furthermore, in nearly all years, students with asthma were significantly more likely to smoke cigarettes and cigars and to use marijuana than students without asthma. The greater use of these products among students with asthma is of public health concern because inhalation of tobacco smoke is a known asthma trigger [1,12] and marijuana smoke can also have adverse respiratory effects [6–8,12].

The observed decline in cigarette and cigar smoking is encouraging given that the U.S. Surgeon General has concluded that combustible tobacco products are responsible for the overwhelming burden of death and disease from tobacco use in the United States [2]. However, consistent with previous research [14,16], the findings from this study reinforce the importance of continued efforts to further reduce tobacco use among all youth, but particularly among those with asthma who could experience exacerbated symptoms by smoking combustible tobacco products such as cigarettes or cigars [1,2,12]. The continued regulation of the manufacturing, distribution, and marketing of tobacco products by the Food and Drug Administration, in coordination with the implementation of proven population-based strategies could help further reduce all forms of tobacco use, including combustible tobacco products, among U.S. youth with and without asthma [2,20]. Strategies shown to be effective in preventing and reducing youth tobacco use include increasing the price of tobacco products, protecting people from secondhand exposure to combustible tobacco smoke and e-cigarette aerosol, implementing advertising and promotion restrictions and national public education media campaigns, and raising the minimum age of purchase for tobacco products to 21 years [2,20].

Marijuana use remained stable during 2003–2017, with an estimated 1 in 4 high school students with asthma using marijuana during this period. Much is still to be learned about the respiratory health effects of marijuana use [6]. The limited evidence about the health effects of marijuana use may influence public knowledge about the risks of marijuana use, and may partly explain continued high rates of use among students with and without asthma. However, a growing body of evidence suggests that smoking marijuana affects lung function and is harmful to the lungs [6–8,12]. Smoke from marijuana contains many of the same toxins, irritants, and carcinogens as tobacco smoke [11]. Although marijuana and tobacco products differ in multiple ways, including established health risks, the strategies used to regulate these products are often similar [21].

Some variation in tobacco and marijuana use by asthma status was observed across demographic subgroups. For example, current cigarette, cigar, and marijuana use was higher among female students with asthma than among female students without asthma. Moreover, frequency of use was higher among students with asthma compared to those without asthma. Although higher rates of cigarette, cigar, and marijuana use among students with asthma compared to those without was not found for all demographic subgroups, for no group was use of these substances lower among students with asthma. These findings highlight the importance of efforts to prevent and reduce the use of these products among youth. For example, health care providers could inquire about the use of tobacco and marijuana among all of their pediatric patients, irrespective of whether they have asthma. The American Academy of Pediatrics recommends providers both “include tobacco use prevention as part of anticipatory guidance” [22, p. 1010], and “offer tobacco dependence treatment and/or referral to adolescents who want to stop smoking” [22, p. 1012]. Similarly, others have suggested that health care providers should question their patients with asthma about marijuana use, and advise those patients who use marijuana to avoid its use [7,12]. While students are at school, school nurses serve as health care managers [23,24], helping students manage their chronic conditions [23,24]. Thus, in some cases, it may be the school nurse who identifies students with asthma who smoke tobacco or use marijuana. When warranted, school nurses are in a position to either provide direct care in the form of cessation guidance or refer students to community or health care services for cessation treatment [23,25].

This study is subject to some limitations. First, these data apply only to youth who attend school and, therefore are not representative of all persons in this age group. However, the extent of such bias would be expected to be minimal given that nationwide, approximately 5% of persons aged 16–17 years in 2013 were not enrolled in high school [26]. Second, medical records were not used to confirm self-reported asthma status, and the extent of under-reporting or over-reporting of current use of cigarettes, cigars, and marijuana and asthma status cannot be determined. Third, although it is possible that some students who used marijuana did not smoke it, an estimated 90% of marijuana users report that combustion is their primary mode of use [9,10]. Finally, blunts, emptied cigars that have been partly or completely refilled with marijuana [11], are a common way youth use marijuana [27,28]. It is possible that some misclassification of use could have occurred depending on whether students who used blunts considered the product to be marijuana, a cigar, or both. The use of electronic vapor products was excluded from this analysis because those data were available for only 2015 and 2017; therefore, it was not possible to assess trends in use. Additionally, given that these products have been available for a relatively short time period, existing research on potential health effects is limited, particularly with regard to asthma. Accordingly, future research on the potential impact of electronic vapor product use on the exacerbation of asthma would be beneficial, including whether students with asthma are more prone than their peers to use those products.

## Conclusion

The prevalence of cigarette and cigar smoking declined during 2003–2017 among students with asthma at a rate similar to that of students without asthma; however, for nearly every year the prevalence of each was higher among students with asthma than without asthma. The prevalence of marijuana use did not change during 2003–2017 among either group, but the prevalence was higher among students with asthma than among students without asthma every year except 2005. These findings highlight the importance of sustained population-based efforts, including school health services, to prevent and reduce the use of cigarettes, cigars, and marijuana among youth, particularly among students with asthma [2,3,8].

## Figures and Tables

**Table 1.** Prevalence of current cigarette smoking, current cigar smoking, and current marijuana use by asthma status among sex, race/ethnicity, and grade subgroups—2017 National Youth Risk Behavioral Survey.

	Prevalence of Current Smoking^a^		Prevalence of Current Cigar Smoking^b^		Prevalence of Current Marijuana Use^c^	
	With Asthma^d^ % (95% CI^e^)	Without Asthma % (95% CI)	With Asthma % (95% CI)	Without Asthma % (95% CI)	With Asthma % (95% CI)	Without Asthma % (95% CI)
Total	10.7 (8.1–14.0)	8.2 (6.8–9.8)*	9.7 (8.0–11.7)	7.3 (6.5–8.2)*	23.4 (21.1–25.9)	18.5 (16.8–20.3)*
Sex						
Female	10.1 (7.1–14.1)	7.1 (5.6–8.8)*	7.4 (5.7–9.5)	4.7 (3.9–5.7)*	24.7 (21.5–28.2)	18.0 (15.7–20.6)*
Male	11.0 (8.6–14.1)	9.4 (7.8–11.2)	11.8 (9.3–14.8)	9.9 (8.8–11.1)	22.0 (18.7–25.6)	18.9 (17.1–20.8)
Race/ethnicity						
White^f^	13.7 (10.0–18.4)	10.3 (8.4–12.5)*	10.8 (8.3–13.8)	8.4 (7.2–9.8)	21.2 (18.2–24.5)	16.6 (14.3–19.2)*
Black^f^	5.6 (3.1 −9.9)	3.2 (1.7–5.7)	8.6 (5.7–12.8)	5.6 (4.3–7.1)	26.5 (21.9–31.7)	24.1 (20.8–27.7)
Hispanic	8.6 (6.3–11.5)	6.6 (5.2–8.4)	8.3 (6.1–11.3)	5.7 (4.6–6.9)	29.3 (24.0–35.1)	21.7 (18.2–25.7)*
Grade						
9	6.2 (3.7–10.2)	4.7 (3.4–6.5)	6.1 (4.1–9.1)	4.3 (3.3–5.6)	16.5 (12.2–21.9)	11.9 (10.2–13.9)*
10	9.8 (7.1–13.3)	7.0 (5.3–9.0)	6.5 (4.5–9.2)	5.0 (4.2–6.1)	22.1 (19.1 −25.4)	17.6 (15.4–20.0)*
11	12.8 (9.2–17.6)	8.3 (6.4–10.8)*	11.7 (8.8–15.4)	8.2 (6.8–9.9)*	26.4 (22.2–31.1)	21.3 (18.8–23.9)*
12	14.1 (9.6–20.2)	13.2 (10.6–16.2)	14.3 (11.5–17.6)	11.8 (10.0–13.9)	29.8 (26.2–33.8)	23.8 (20.8–27.2)*

* Significant difference between students with and without asthma (*p* < .05).

a Smoked cigarettes on one or more days during the 30 days before the survey.

b Smoked cigars (cigars, cigarillos, or little cigars) on one or more days during the 30 days before the survey.

c Used marijuana one or more times during the 30 days before the survey.

d A doctor or nurse ever told them they have asthma.

e Confidence interval.

f Non-Hispanic.

**Table 2.** Number of days of smoking cigarettes and cigars and number of times using marijuana among students with and without asthma—2017 National Youth Risk Behavioral Survey.

	Asthma status	
	With asthma^a^ % (95% CI^b^)	Without asthma % (95% CI)
Days of cigarette smoking^c^		
0 days	89.3 (85.9–92.0)	91.8 (90.1–93.2)*
1 or 2 days	2.9 (2.3–3.5)	3.0 (2.5–3.5)
3 to 5 days	1.7 (1.3–2.4)	1.2 (0.9–1.6)*
6 to 9 days	1.2 (0.7–2.0)	0.9 (0.7–1.3)
10 to 19 days	0.9 (0.6–1.5)	0.9 (0.6–1.1)
20 to 29 days	0.7 (0.3–1.5)	0.6 (0.4–0.8)
All 30 days	3.2 (2.0–5.1)	1.7 (1.2–2.4)*
Days of cigar smoking^d^		
0 days	90.3 (88.2–92.1)	92.7 (91.8–93.5)*
1 or 2 days	3.5 (2.7–4.4)	3.2 (2.8–3.8)
3 to 5 days	1.7 (1.2–2.5)	1.5 (1.2–1.9)
6 to 9 days	1.6 (1.0–2.4)	0.9 (0.7–1.2)*
10 to 19 days	0.8 (0.5–1.2)	0.6 (0.5–0.8)
20 to 29 days	0.5 (0.2–0.9)	0.3 (0.1–0.4)
All 30 days	1.7 (1.0–2.7)	0.7 (0.5–1.0)*
Times used marijuana^c^		
0 times	76.6 (74.0–78.9)	81.5 (79.7–83.3)*
1 or 2 times	7.1 (6.0–8.4)	6.6 (5.9–7.3)
3 to 9 times	5.6 (4.7–6.6)	4.8 (4.1–5.5)
10–19 times	2.9 (2.1–4.1)	2.1 (1.6–2.6)
20–39 times	2.7 (2.0–3.5)	1.5 (1.2–1.9)*
40 or more times	5.2 (3.9–6.9)	3.5 (2.8–4.4)*

* Significant difference between students with and without asthma (*p* < .05).

a A doctor or nurse ever told them they have asthma.

b Confidence interval.

c During the 30 days before the survey.

d Cigars, cigarillos, or little cigars, during the 30 days before the survey.

**Table 3.** Trends in the prevalence of current cigarette smoking, current cigar smoking, and current marijuana use among students with and without asthma—National Youth Risk Behavioral Surveys, 2003–2017.

	Prevalence of current cigarette smoking^a^		Prevalence of current cigar smoking^b^		Prevalence of current marijuana use^c^	
	With Asthma^d^ % (95% CI^e^)	Without Asthma % (95% CI)	With Asthma % (95% CI)	Without Asthma % (95% CI)	With Asthma % (95% CI)	Without Asthma % (95% CI)
**Year**						
2003	25.2 (22.3–28.5)	22.1 (19.9–24.4)*	15.8 (13.8–18.0)	13.8 (12.6–15.2)*	26.4 (23.0–30.1)	22.3 (20.0–24.9)*
2005	24.3 (21.7–27.1)	22.6 (20.2–25.2)	15.9 (13.7–18.4)	13.5 (12.1–15.2)*	21.9 (19.3–24.6)	19.7 (18.1–21.4)
2007	21.1 (18.2–24.4)	19.6 (17.3–22.2)	14.7 (12.3–17.5)	13.1 (11.6–14.8)	21.7 (19.0–24.6)	19.1 (17.3–21.0)*
2009	22.6 (20.2–25.1)	18.5 (17.0–20.1)*	16.6 (15.0–18.4)	13.2 (11.9–14.7)*	23.4 (21.3–25.6)	20.0 (18.6–21.5)*
2011	21.3 (19.2–23.5)	17.5 (16.0–19.0)*	15.2 (13.3–17.2)	12.6 (11.6–13.6)*	26.5 (24.5–28.6)	22.0 (20.3–23.8)*
2013	17.8 (15.0–20.9)	15.1 (13.0–17.4)*	14.7 (12.8–16.9)	12.0 (10.9–13.2)*	26.6 (23.3–30.2)	22.3 (20.5–24.4)*
2015	13.4 (11.2–16.1)	10.1 (8.6–11.7)*	13.0 (10.9–15.5)	9.4 (8.1–10.9)*	25.9 (23.5–28.3)	20.0 (17.3–23.1)*
2017	10.7 (8.1–14.0)	8.2 (6.8–9.8)*	9.7 (8.0–11.7)	7.3 (6.5–8.2)*	23.4 (21.1–25.9)	18.5 (16.8–20.3)*
Linear trend^f^	*p* < 0.01	*p* < 0.01	*p* < 0.01	*p* < 0.01	*P* = 0.24	*P* = 0.67
Interaction^g^	*P* = 0.07		*P* = 0.08		*P* = 0.09	

* Significant difference between students with and without asthma (*p* < .05).

a Smoked cigarettes on one or more days during the 30 days before the survey.

b Smoked cigars (cigars, cigarillos, or little cigars) on one or more days during the 30 days before the survey.

c Used marijuana one or more times during the 30 days before the survey.

d A doctor or nurse ever told them they have asthma.

e Confidence interval.

f Logistic regression model testing linear trends, controlling for sex, grade, race/ethnicity.

g The interaction term tests whether the trends in current use of cigarettes, cigars, and marijuana differ by asthma status.

## Abbreviations

YRBS: Youth Risk Behavior Survey
CDC: Centers for Disease Control and Prevention
